# CXCL16 suppresses liver metastasis of colorectal cancer by promoting TNF-α-induced apoptosis by tumor-associated macrophages

**DOI:** 10.1186/1471-2407-14-949

**Published:** 2014-12-15

**Authors:** Ji-Ye Kee, Aya Ito, Shozo Hojo, Isaya Hashimoto, Yoshiko Igarashi, Koichi Tsuneyama, Kazuhiro Tsukada, Tatsuro Irimura, Naotoshi Shibahara, Ichiro Takasaki, Akiko Inujima, Takashi Nakayama, Osamu Yoshie, Hiroaki Sakurai, Ikuo Saiki, Keiichi Koizumi

**Affiliations:** Division of Pathogenic Biochemistry, University of Toyama, Toyama, 930-0194 Japan; Division of Kampo Diagnostics, Institute of Natural Medicine, University of Toyama, 2630 Sugitani, Toyama, 930-0194 Japan; Department of Surgery (II), Faculty of Medicine, University of Toyama, Toyama, 930-0194 Japan; Department of Pathology (I), Faculty of Medicine, University of Toyama, Toyama, 930-0194 Japan; Institute for Medical Innovation, St Luke’s International Medical Center, Tokyo, 104-8560 Japan; Division of Chemotherapy, Kinki University School of Pharmaceutical Sciences, Osaka, 577-8502 Japan; Department of Microbiology, Kinki University Faculty of Medicine, Osaka, 589-8511 Japan; Department of Cancer Cell Biology, Graduate School Medicine and Pharmaceutical Sciences, University of Toyama, Toyama, 930-0194 Japan; Molecular Genetics Research Center, University of Toyama, Toyama, 930-0194 Japan

**Keywords:** CXCL16, IRF8, TNF-α, Apoptosis, Colorectal liver metastasis

## Abstract

**Background:**

Inhibition of metastasis through upregulation of immune surveillance is a major purpose of chemokine gene therapy. In this study, we focused on a membrane-bound chemokine CXCL16, which has shown a correlation with a good prognosis for colorectal cancer (CRC) patients.

**Methods:**

We generated a CXCL16-expressing metastatic CRC cell line and identified changes in TNF and apoptosis-related factors. To investigate the effect of CXCL16 on colorectal liver metastasis, we injected SL4-Cont and SL4-CXCL16 cells into intraportal vein in C57BL/6 mice and evaluated the metastasis. Moreover, we analyzed metastatic liver tissues using flow cytometry whether CXCL16 expression regulates the infiltration of M1 macrophages.

**Results:**

CXCL16 expression enhanced TNF-α-induced apoptosis through activation of PARP and the caspase-3-mediated apoptotic pathway and through inactivation of the NF-κB-mediated survival pathway. Several genes were changed by CXCL16 expression, but we focused on IRF8, which is a regulator of apoptosis and the metastatic phenotype. We confirmed CXCL16 expression in SL4-CXCL16 cells and the correlation between CXCL16 and IRF8. Silencing of IRF8 significantly decreased TNF-α-induced apoptosis. Liver metastasis of SL4-CXCL16 cells was also inhibited by TNF-α-induced apoptosis through the induction of M1 macrophages, which released TNF-α. Our findings suggest that the accumulation of M1 macrophages and the enhancement of apoptosis by CXCL16 might be an effective dual approach against CRC liver metastasis.

**Conclusions:**

Collectively, this study revealed that CXCL16 regulates immune surveillance and cell signaling. Therefore, we provide the first evidence of CXCL16 serving as an intracellular signaling molecule.

**Electronic supplementary material:**

The online version of this article (doi:10.1186/1471-2407-14-949) contains supplementary material, which is available to authorized users.

## Background

Colorectal cancer (CRC) is the most commonly diagnosed cancer worldwide [1]. Metastasis is the major cause of CRC mortality, and surgery is the only feasible therapy with very low mortality. However, only 10-20% of CRC patients with liver metastasis are candidates for surgery [2]. Consequently, gene therapy is viewed as a promising treatment strategy that can complement the use of existing chemotherapy, radiation therapy and surgery strategies in these patients [3].

Chemokines are a family of small cytokines that function as chemoattractants for several immune effector cell types [4]. Recent studies demonstrated that various chemokines have the potential to suppress tumor growth and metastasis [4, 5]. One unique membrane-bound chemokine is chemokine (C-X-C motif) ligand 16 (CXCL16), which exists as a transmembrane form (TM-CXCL16) as well as a soluble form (sCXCL16) that is cleaved by proteolytic enzymes [6–11]. TM-CXCL16 can function as a cell adhesion molecule for its receptor cells that express CXCR6, such as activated CD8 T cells and natural killer T cells (NKT cells), whereas sCXCL16 is a chemoattractant for CXCR6-expressing cells [12, 13]. Recently, the chemokine/receptor axis has been shown to play a critical role in tumor progression and metastasis [14]. With respect to the CXCL16/CXCR6 axis, we were the first to report that CXCL16 expression by tumor cells enhances the recruitment of tumor-infiltrating lymphocytes, thereby bringing about a better prognosis for CRC patients [15]. Our studies have confirmed the expression of CXCL16 in various cancer cell lines and tumor tissues [16–23], indicating that CXCL16 might serve as a useful biomarker for various types of cancer.

Macrophages function in both innate and adaptive immunity as immune regulatory cells. In particular, tumor-associated macrophages (TAMs) play an important role in the progression and metastasis of cancer [24]. TAMs have been typically defined as M1- and M2-type macrophages. M1 macrophages are potent effector cells that induce Th1 responses such as cytotoxicity against microorganisms and cancer cells and enhancement of pro-inflammatory cytokine production [25, 26]. Tumor-infiltrating macrophages are reported to reduce the development of peritoneal colorectal carcinoma metastasis [27], while liver macrophages exert a protective function against cancer cells and inhibit liver metastasis due to their cytotoxic action against cancer cells through the production of tumor necrosis factor-alpha (TNF-α) [28–30].

TNF-α is typically produced by macrophages that show antitumor activity [31]. TNF-α stimulates intracellular signaling pathways involving caspases, mitogen-activated protein kinases (MAPKs), and nuclear factor kappa B (NF-κB). Activation of the caspases involved in apoptosis results in the cleavage of a large number of nuclear proteins that are essential for apoptosis-associated chromatin margination, DNA fragmentation, and nuclear collapse [32].

Interferon regulatory factor 8 (IRF8) is expressed in cells of myeloid and lymphoid lineages and serves as a key transcription factor [33, 34]. IRF8 has been shown to regulate Fas-mediated apoptosis in myeloid cells and soft tissue sarcoma cells [35, 36]. Deficiency of IRF8 in metastatic human CRC cells leads to decreased spontaneous apoptosis and enhanced resistance to the induction of extrinsic apoptosis [37, 38]. IRF8 is also an essential regulator of the apoptosis pathway and a suppressor of metastasis [39].

In a previous study, we identified genes which expression was changed by CXCL16 expression in metastatic CRC cells. Among these genes, the expression of IRF8 was correlated with CXCL16 expression and showed sensitivity to TNF-α-induced apoptosis. In addition, CXCL16 expression induced the infiltration of M1 macrophages into metastatic tumors and inhibited liver metastasis by releasing TNF-α, thereby inducing the apoptosis of CXCL16-expressing metastatic CRC cells.

## Methods

### Antibodies and reagents

Anti-phospho p65 (Ser-536), Akt (Ser-473), JNK (Thr183/Tyr185), ERK (Thr-202, Tyr-204), p38 (Thr-180/Tyr-182), PARP (46D11) and caspase-3 (8G10) antibodies were purchased from Cell Signaling Technology (Danvers, MA, USA). Antibodies against Akt (C-20), p38 (C-20), JNK (FL), ERK1 (C-16), p65 (C-20-G), IκBα (L35A5), IRF8 (C-19) and β-actin (C-11) were purchased from Santa Cruz Biotechnology (Santa Cruz, CA, USA). CXCL16 antibody and recombinant mouse TNF-α were obtained from R&D Systems (Minneapolis, MN, USA). Mouse TNF-α neutralizing antibody and 2-chloroadenosine were purchased from eBioscience (San Diego, CA, USA) and Sigma (St Louis, MO, USA), respectively.

### Cell culture

The mouse colon carcinoma cell lines, colon 38 and colon 38 SL4 (SL4), were maintained in a 1:1 mixture of Dulbecco’s modified Eagle’s medium and Ham’s F-12 medium (DMEM/F12; Invitrogen, Carlsbad, CA, USA). The mouse leukemic monocyte macrophage cell line, RAW 264.7, was maintained in DMEM. The media contained 10% heat-inactivated fetal calf serum (FCS), 100 units/ml penicillin, and 100 μg/ml streptomycin.

### Generation of CXCL16-expressing CRC cell line

We generated pcDNA3.1 (+)-CXCL16, which was based on the pcDNA 3.1 (+) expression vector (Life Technologies Japan Ltd., Tokyo, Japan), to express the mouse membrane-bound CXCL16. Nucleofector (Amaxa, Gaithersburg, MD, USA) was used to transfect colon 38 SL4 cells with pcDNA3.1 (+)-CXCL16 or the empty vector. DNA was adjusted to 1 μg with the empty vector. After transfection, CXCL16-positive colon 38 SL4 cells were selected using the antibiotic G418 (Invitrogen). Cells stably expressing CXCL16 (SL4-CXCL16) and control cells (SL4-Cont) were maintained in DMEM/F12 supplemented with 10% FCS and antibiotics.

### WST-8 assay

Cell viability was quantified using the cell proliferation reagent WST-8 (2-(2-methoxy-4-nitrophenyl)-3-(4-nitrophenyl)-5-(2, 4-disulfophenyl)-2H-tetrazolium, monosodium salt) (Dojindo, Kumamoto, Japan). Cells were seeded in 96-well microplates (2 × 10^3^ cells) and then TNF-α was added. After 24-48 h incubation, WST-8 solution was added and the absorbance was measured at 450 nm.

### Microarray analysis

Gene expression was analyzed using a GeneChip1 system with the mouse Expression Array 430.2 (Affymetrix, Santa Clara, CA, USA). Samples were prepared for array hybridization following the manufacturer’s instructions. In brief, 2 μg total RNA was used to synthesize double-stranded cDNA with a GeneChip1 Expression 30-Amplification Reagents One-Cycle cDNA Synthesis Kit (Affymetrix). Subsequently, biotin-labeled cRNA was synthesized from cDNA using the GeneChip1 Expression 30-Amplification Reagents for IVT Labeling (Affymetrix). Following fragmentation, biotinylated cRNA was hybridized to arrays at 45°C for 16 h. The arrays were washed, stained with streptavidin–phycoerythrin, and scanned with a probe array scanner. The scanned chip was analyzed using GeneChip Microarray Suite software (Affymetrix). Hybridization intensity data were converted into a presence/absence call for each gene, and changes in gene expression between experiments were detected via comparison analysis. Data were further analyzed using GeneSpring (Silicon Genetics, Redwood City, CA, USA). The GeneSpring Filter on the Volcano Plot tool was implemented to obtain a list of differentially expressed significant genes. A fold change value greater (upregulated) or less than 2 (downregulated) was considered biologically important. The statistical significance of the fold change was calculated for 2 groups by Student’s *t*-test and *P* values less than 0.05 were considered significant.

### Reverse-transcription PCR (RT-PCR)

Total RNA was extracted using an RNeasy Mini Kit (Qiagen, Valencia, CA, USA) according to the manufacturer’s directions. First-strand cDNA was prepared from an RNA template (2 μg) using oligo (dT) 18 primer and SuperScript III reverse transcriptase (Invitrogen). Reverse transcription was performed at 42°C for 50 min and then at 70°C for 15 min. PCR amplification was performed by denaturation at 94°C for 5 s, annealing at 60°C for 5 s, and extension at 72°C for 10 s for 28 cycles using a SappireAmp Fast PCR Master Mix (TaKaRa, Kyoto, Japan). Forward/reverse RT-PCR primer pairs for mouse cDNAs were as follows: CD11b (5′-ACACCATCGCATCTAAGCCA-3′/5′-GAACATCACCACCAAGCCAA-3′); CD11c (5′-CTTCTGCTGTTGGGGTTTGT-3′/5′-CACGATGTCTTGGTCTTGCT-3′); F4/80 (5′-CTTGCTGGAGACTGTGGAA-3′/5′-TGGATGTGCTGGAGGGTAT-3′); TNF-α (5′-GATCTCAAAGACAACCAACTAGTG-3′/5′-CTCCAGCTGGAAGACTCCTCCCAG-3′); GAPDH (5′-TGAAGGTCGGAGTCAACGGATTTGGT-3′/5′-CATGTGGGCCATGAGGTCCACCAC-3′). PCR products were electrophoresed on 1.5% agarose gels and stained with SYBR green. Images were acquired by Gel Doc EZ Imager (Bio-Rad, Hercules, CA, USA).

### Real-time RT-PCR (qRT-PCR)

The cDNAs were amplified using FastStart Essential DNA Green Master (Roche, Pleasanton, CA, USA). Forward/reverse RT-PCR primer pairs for mouse cDNAs were as follows: CXCL16 (5′-TGAACTAGTGGACTGCTTTGAGC-3′/5′-GCAAATGTTTTTGGTGGTGA-3′); IRF8 (5′-GAGCCAGATCCTCCCTGACT-3′/5′-GGCATATCCGGTCACCAGT-3′); CD11b (5′-AAGGATGCTGGGGAGGTC-3′/5′-GTCATAAGTGACAGTGCTCTGGAT-3′); CD11c (5′-GAGCCAGAACTTCCCAACTG-3′/5′-TCAGGAACACGATGTCTTGG-3′); F4/80 (5′-GGAGGACTTCTCCAAGCCTATT-3′/5′-AGGCCTCTCAGACTTCTGCTT-3′); TNF-α (5′-CTGTAGCCCACGTCGTAGC-3′/5′-TTGAGATCCATGCCGTTG-3′); β-actin (5′-CTAAGGCCAACCGTGAAAAG-3′/5′-ACCAGAGGCATACAGGGACA-3′). Real-time quantitative RT-PCR (qRT-PCR) was performed using a Lightcycler nano system (Roche). The gene expression data were normalized to the β-actin. The relative expression levels of genes were measured according to the formula 2^-Δ*Ct*^, where Δ*Ct* is the difference in threshold cycle values between the targets and β-actin.

### Transfection with small interfering RNA (siRNA)

Mouse IRF8 siRNA and control siRNA were purchased from Santa Cruz Biotechnology. Mouse CXCL16 siRNA was purchased from Ambion Life Technologies (Carlsbad, CA, USA). SL4 cells were transfected with siRNAs at a final concentration of 20 nM (si-IRF8) or 100 nM (si-CXCL16) using Lipofectamine reagents (Invitrogen). After 5 h, the medium was changed to normal medium and cells were cultured for a further 24 h.

### Annexin V assay

The Annexin V assay was carried out using Annexin V Apoptosis Detection Kit I (BD Biosciences, San Diego, CA, USA). In brief, harvested cells (1 × 10^6^ cells) were washed twice with phosphate-buffered saline (PBS) and cells were resuspended in 1 ml Annexin V binding buffer. Then, 100 μl of the solution was transferred to a 5 ml culture tube and labeled with 2 μl titrated FITC Annexin V and Propidium Iodide Staining Solution (PI). The cells were vortexed and incubated for 15 min at room temperature in the dark. The volume was then made up to 500 μl and the cells were analyzed with the FACSCalibur system (BD Biosciences).

### Western blot analysis

Cells were harvested, plated on a 6 cm dish (1 × 10^6^ cells) and stimulated with TNF-α (10 ng/ml). Whole-cell lysates were prepared with lysis buffer (25 mM HEPES pH 7.7, 0.3 M NaCl, 1.5 mM MgCl_2_, 0.2 mM EDTA, 10% Triton X-100, 20 mM β-glycerophosphate, 1 mM sodium orthovanadate, 1 mM dithiothreitol (DTT), 10 μg/ml aprotinin, and 10 μg/ml leupeptin). Cell lysates were collected from the supernatant after centrifugation for sulfate-polyacrylamide gel electrophoresis and transferred to an immobilon-P-nylon membrane (Millipore, Bedford, MA, USA). The membrane was blocked with Block Ace (Dainippon Pharmaceutical, Osaka, Japan) and probed with primary antibodies. The antibodies were detected using horseradish peroxidase-conjugated anti-rabbit and anti-mouse immunoglobulin G (Dako, Glostrup, Denmark) and blots were detected using the ECL system (GE Healthcare, Piscataway, NJ, USA).

### Experimental liver metastasis

For experimental liver metastasis, colon 38 SL4 cells (7.5 × 10^4^ cells/200 μl PBS) were injected into the intraportal vein of mice. The animals were sacrificed 17 days later and the increases in liver weight and the numbers of tumor colonies in the livers were measured to evaluate tumor metastasis. All experimental protocols were approved by the Laboratory Animal Care and Use Committee of Toyama University and were performed according to the Guidelines for the Care and Use of Laboratory Animals of Toyama University. Five-week-old female C57BL/6 mice (supplied by Japan SLC, Inc., Hamamatsu, Japan) were used in all experiments. Room temperature was maintained between 23 and 25°C, and relative humidity was maintained between 45 and 60%. The institutional laboratory housing the cages provided a 12-hour light cycle.

### Statistical analysis

Data were analyzed for statistical significance using Student’s *t*-test. *P* < 0.05 was considered significant. The mean and SD were calculated for all variables.

## Results

### CXCL16 expression enhances the sensitivity of metastatic CRC cells to TNF-α-induced apoptosis

**Figure 1:**
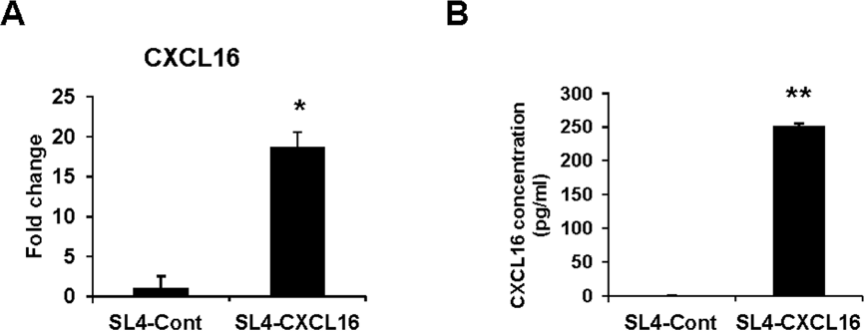
**Establishment of a cell line that stably overexpressed CXCL16. (A)** The mRNA level of CXCL16 was analyzed by qRT-PCR. SL4-Cont and SL4-CXCL16 cells were cultured for 24 h and lysed to extract total RNA. These data were normalized to GAPDH and expressed relative to the SL4-Cont levels, which were assigned a value of 1. **(B)** Protein level of CXCL16. Cells were seeded in a 24-well plate and the supernatant was collected after 24 h for ELISA. *P <0.05, **P <0.01. All experiments were repeated at least three times.

**Figure 2:**
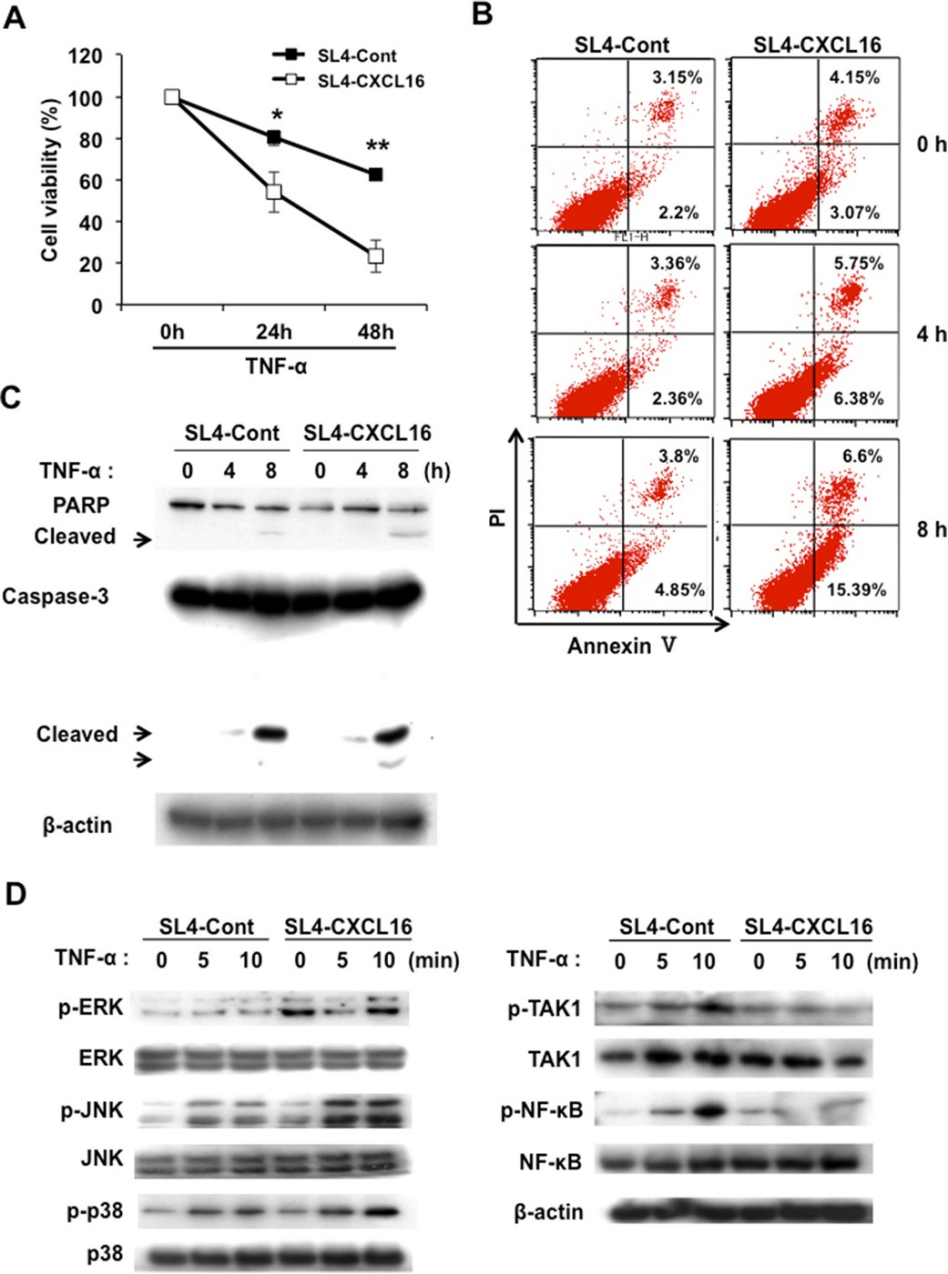
**CXCL16 overexpression sensitizes SL4 cells to TNF-α-induced cell death. (A)** Viability of SL4-Cont and SL4-CXCL16 cells following TNF-α stimulation. Cells were seeded in 96-well plates (2 × 10^3^ cells) stabilized for 1 h and stimulated by TNF-α (10 ng/ml) for 24–48 h. *P <0.05, **P <0.01. **(B)** Annexin V assay. Cells were seeded in 6-well plates (2 × 10^5^ cells) and treated with TNF-α (10 ng/ml) for 0-8 h. **(C)** Effects of CXCL16 expression on the TNF-α-induced apoptotic pathway in SL4 cells. **(D)** Effects of CXCL16 expression on the TNF-α-induced NF-κB and MAPK signaling pathways in SL4 cells. β-actin was used as a normalization control. All experiments were repeated at least three times.

We established a stable expression of CXCL16 in SL4, a metastatic mouse CRC cell line, and confirmed its expression (Figure 1). This cell line was cultured in a polyclonal population with a different expression level of CXCL16 in the antibiotic G418 to maintain the heterozygous characteristics of cancer cells. Membrane-bound CXCL16 was confirmed in almost SL4-CXCL16 cells by flow cytometry (Additional file 1). We next performed microarray analysis to compare gene expressions between SL4-Cont and SL4-CXCL16 cells. Significant differences were noted in the expression of TNF and apoptosis-related factors, whereas no changes were observed in metastatic factors (Table 1). On the other hand, soluble CXCL16 did not affect SL4-CXCL16 cells via the paracrine system because expression of CXCR6 (CXCL16 receptor) was not observed on SL4-CXCL16 cells (Additional file 2). When SL4-Cont and SL4-CXCL16 cells were treated with TNF-α, this induced a time-dependent increase in the death of SL4-CXCL16 cells (Figure 2A). We carried out the Annexin V assay to determine whether the TNF-α-induced cell death was apoptosis. As shown in Figure 2B, apoptosis was greater in TNF-α-treated SL4-CXCL16 cells than in SL4-Cont cells. The TNF-α-induced apoptotic response involved the stimulation of several intercellular signaling pathways [32]. Western blot analysis revealed an increase in the cleavage of PARP and caspase-3 in SL4-CXCL16 cells (Figure 2C). In addition, TNF-α-induced activation of TAK1 and its downstream NF-κB decreased, whereas phosphorylation of ERK and JNK increased in SL4-CXCL16 cells (Figure 2D). These results suggested that CXCL16 expression sensitized metastatic CRC cells to TNF-α-induced apoptosis.

**Table 1:** **Comparison of colon38 SL4-Cont and SL4-CXCL16 cells**

Gene name	Fold change	Gene symbol
**Apoptosis-related factors**		
Irf8	11.89	Interferon regulatory factor 8
Ccnd1	0.45	Cyclin D1
Casp8	0.39	Caspase 8
Bag4	0.33	Bcl2-associated athanogene 4
Bcl2l10	0.33	Bcl2-like 10
Casp9	0.33	Caspase 9
Mapk1	0.33	Mitogen-activated protein kinase 1
Bcl2l2	0.11	BCL2-like 2
**TNF-related factors**		
Ltbr	16.90	Lymphotoxin B receptor
Tnfrsf22	5.83	Tumor necrosis factor receptor superfamily, member 22
Traf4	4.10	TNF receptor associated factor 4
MAP3K7	2.13	Mitogen-activated protein kinase kinase kinase 7
Ttrap	2.01	TRAF and TNF receptor associated protein
Irak1	7.84	Interleukin-1 receptor-associated kinase 1
Traf6	0.44	TNF receptor associated factor 6
Tnfaip2	0.41	Tumor necrosis factor, alpha-induced protein 2
IRAK4	0.36	Interleukin-1 receptor-associated kinase 4
Tnfrsf11a	0.15	Tumor necrosis factor receptor superfamily, member 11a
**Others**		
Tnni2	49.02	Troponin I, skeletal, fast 2
Myh3	34.13	Myosin, heavy polypeptide 3, skeletal muscle embryonic
Pi16	23.76	Peptidase inhibitor 16
Tnni1	20.89	Troponin I, skeletal, slow 1

### Expression of CXCL16 correlates with IRF8 expression in metastatic CRC cells

**Figure 3:**
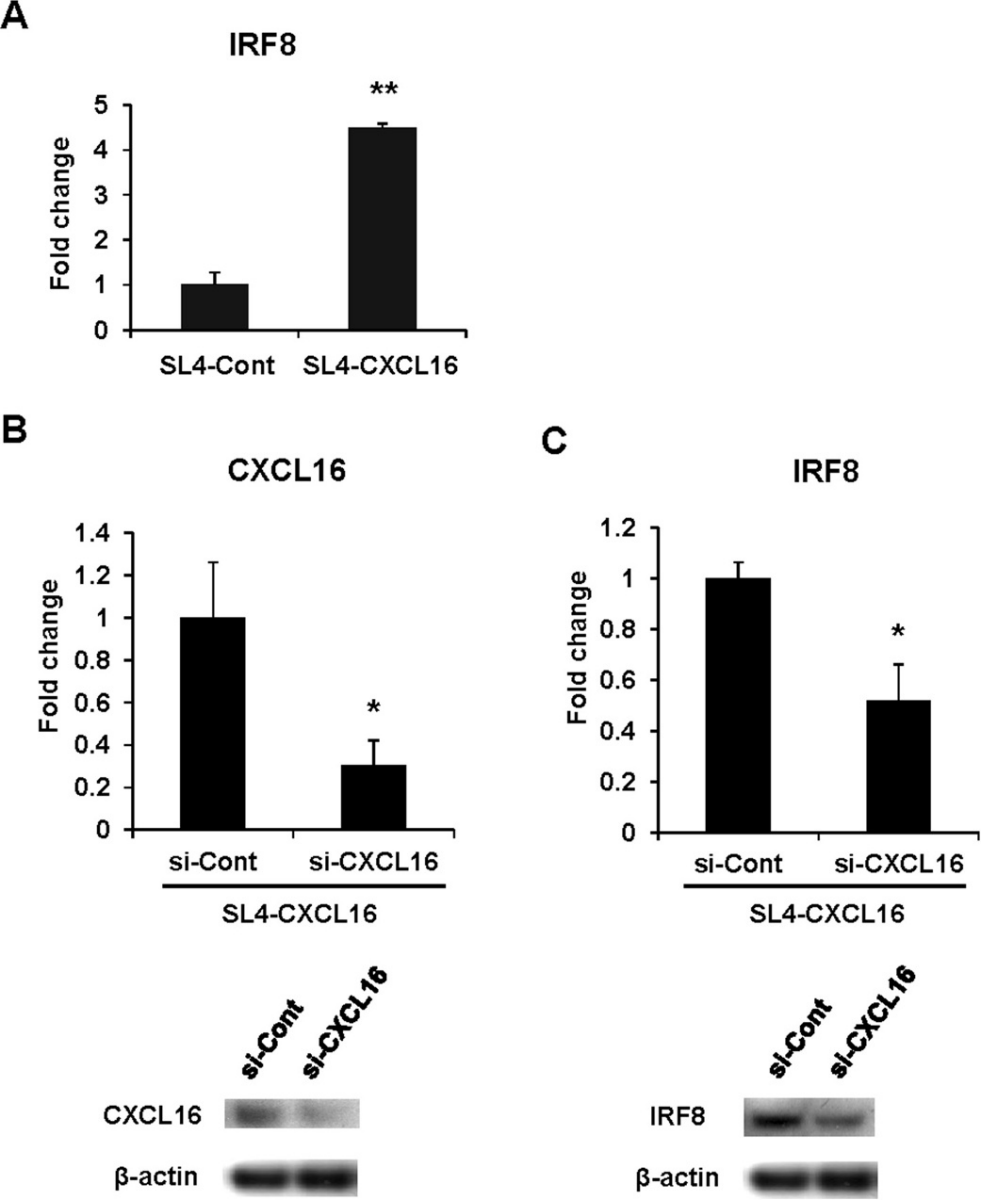
**Correlation of CXCL16 and IRF8 expression in SL4 cells. (A)** Expression of IRF8 in SL4-Cont and SL4-CXCL16 cells cultured for 24 h and analyzed by qRT-PCR. These data were normalized to GAPDH. **(B)** Knockdown of CXCL16 expression. SL4-CXCL16 cells were transfected with siRNA for 36 h to assess the expression of CXCL16 at the mRNA (upper panel) and protein (lower panel) levels. **(C)** Expression levels of IRF8 in SL4-CXCL16 using siRNA for control and CXCL16. β-actin was used as a normalization control. *P <0.05, **P <0.01. All experiments were repeated at least three times.

IRF8 has been shown to be a key regulator of Fas-mediated apoptosis in various cancer cells [34–36] and metastatic phenotypes in CRC cells [37, 38]; however, the correlation between TNF-α-induced apoptosis and IRF8 expression in CRC cells has not been investigated. We confirmed that IRF8 expression was increased in SL4-CXCL16 cells (Figure 3A). In contrast, IRF8 expression was significantly decreased by CXCL16 knockdown (Figure 3C), although CXCL16 expression was not completely silenced by siRNA (Figure 3B). These results indicated that the expression of IRF8 correlates with CXCL16 expression.

### Silencing of IRF8 expression leads to resistance to TNF-α-induced apoptosis

**Figure 4:**
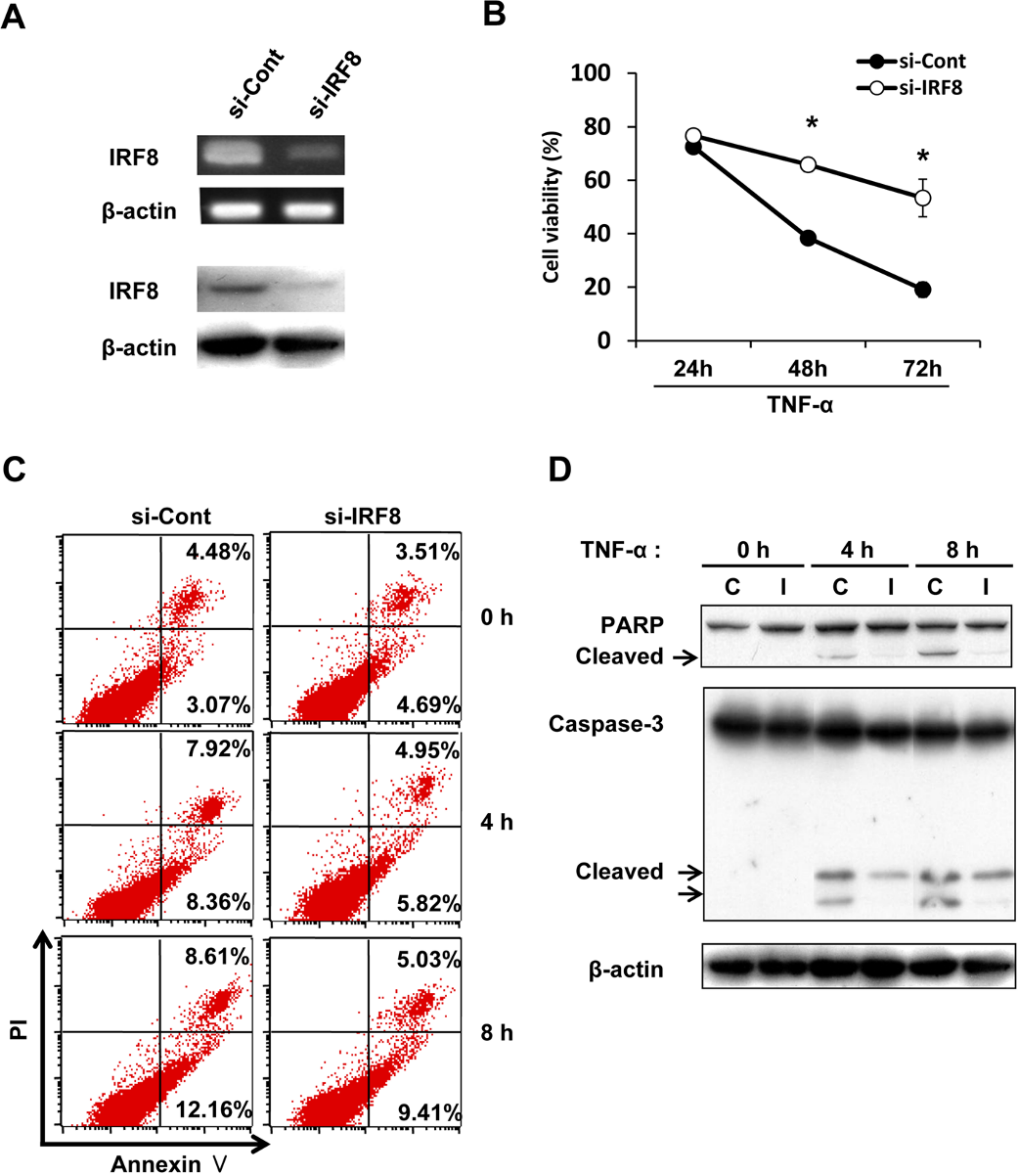
**Silencing of IRF8 expression by siRNA inhibited TNF-α-induced apoptosis in SL4-CXCL16. (A)** Knockdown of IRF8 expression by qRT-PCR and Western blot analysis. **(B)** Viability of IRF8 knockdown cells stimulated with TNF-α. Cells were seeded in 96-well plates (2 × 10^3^ cells) and stimulated with TNF-α (10 ng/ml) for 0–72 h and then viability was measured by the WST-8 assay. *P <0.05. **(C)** Annexin V assay. Cells were seeded in 6-well plates (2 × 10^5^ cells) and treated with TNF-α (10 ng/ml). **(D)** Effects of IRF8 knockdown on TNF-α-induced apoptotic responses. C, si-Control; I, si-IRF8. All experiments were repeated at least three times.

The increased sensitivity to TNF-α-induced apoptosis and IRF8 expression apparent in SL4-CXCL16 cells led us to hypothesize that IRF8 expression was related to sensitivity to TNF-α-induced apoptosis. Regulation of TNF-α-induced apoptosis by IRF8 has not been previously reported, even though it regulates Fas-mediated apoptosis [34–38]. When SL4-CXCL16 cells were transfected with IRF8 siRNA (Figure 4A) and stimulated with TNF-α, TNF-α-induced apoptosis was significantly inhibited in IRF8 knockdown cells compared with control siRNA-transfected cells (si-Cont) (Figure 4B and C). The increased activation of caspase-3 and PARP observed in SL4-CXCL16 cells was also decreased in IRF8 knockdown cells (Figure 4D). These results suggested that CXCL16-mediated upregulation of TNF-α-induced apoptosis in IRF8-sensitized SL4-CXCL16 cells occurred via downstream caspase-3 and PARP signaling.

### Tumor-derived CXCL16 expression inhibited liver metastasis

**Figure 5:**
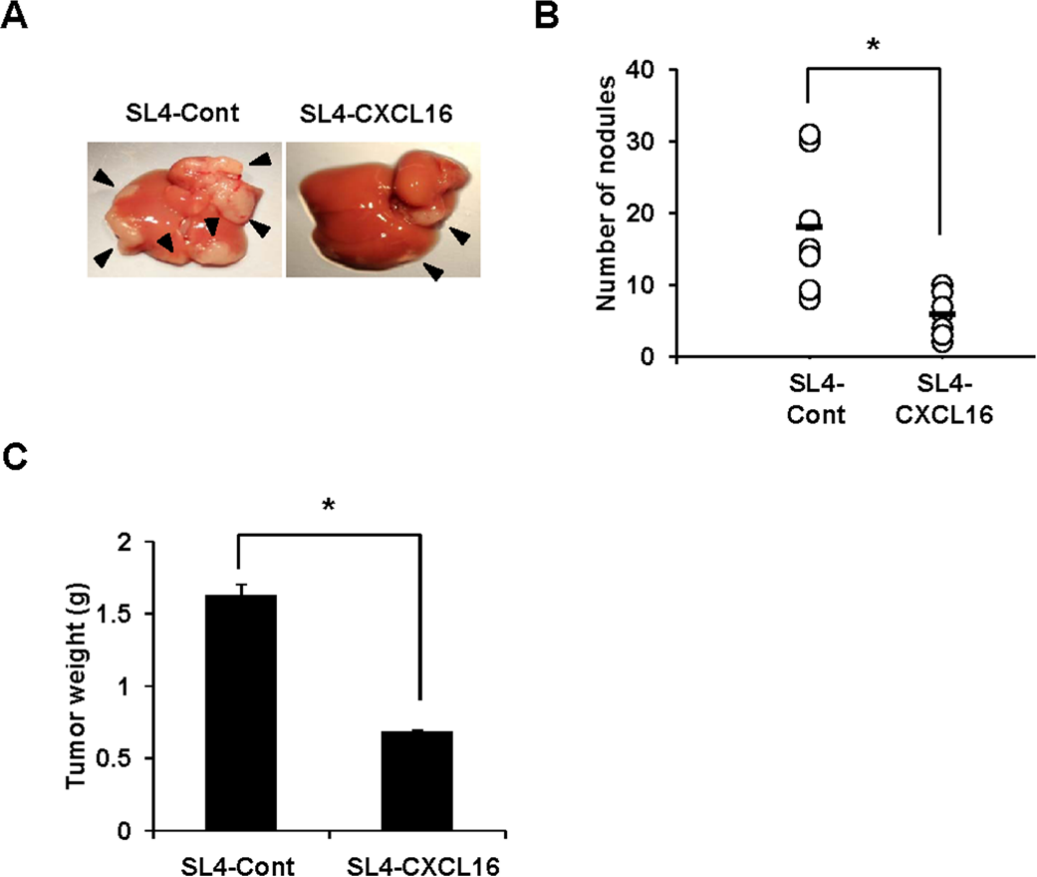
**Tumor-derived CXCL16 inhibited liver metastasis by metastatic CRC cells. (A)** Liver metastasis of SL4 cells. **(B)** Number of nodules on liver metastasis. Horizontal bar is the mean of the data points/group. **(C)** Tumor weight of liver metastasis. Similar results were obtained from three independent experiments (n = 8). *P <0.05. All experiments were repeated at least three times.

The liver is a major metastatic organ of CRC and no specific therapeutic method is available for liver metastasis other than surgical resection [2]. Our finding that CXCL16 expression enhanced the sensitivity of SL4-CXCL16 to TNF-α led us to hypothesize that enhanced sensitivity to TNF-α by CXCL16 expression inhibits in vivo liver metastasis. As shown in Figure 5A, intraportal vein injection of cells into C57BL/6 mice significantly inhibited liver metastasis in the SL4-CXCL16 group compared with the SL4-Cont group (Figure 5B). The difference in tumor weight was evaluated after dividing the resected liver into normal and tumor parts. Tumor weight was significantly lower in the SL4-CXCL16 group than in the SL4-Cont group (Figure 5C).

### Macrophages mediated the inhibition of liver metastasis by CXCL16 through secretion of TNF-α

**Figure 6:**
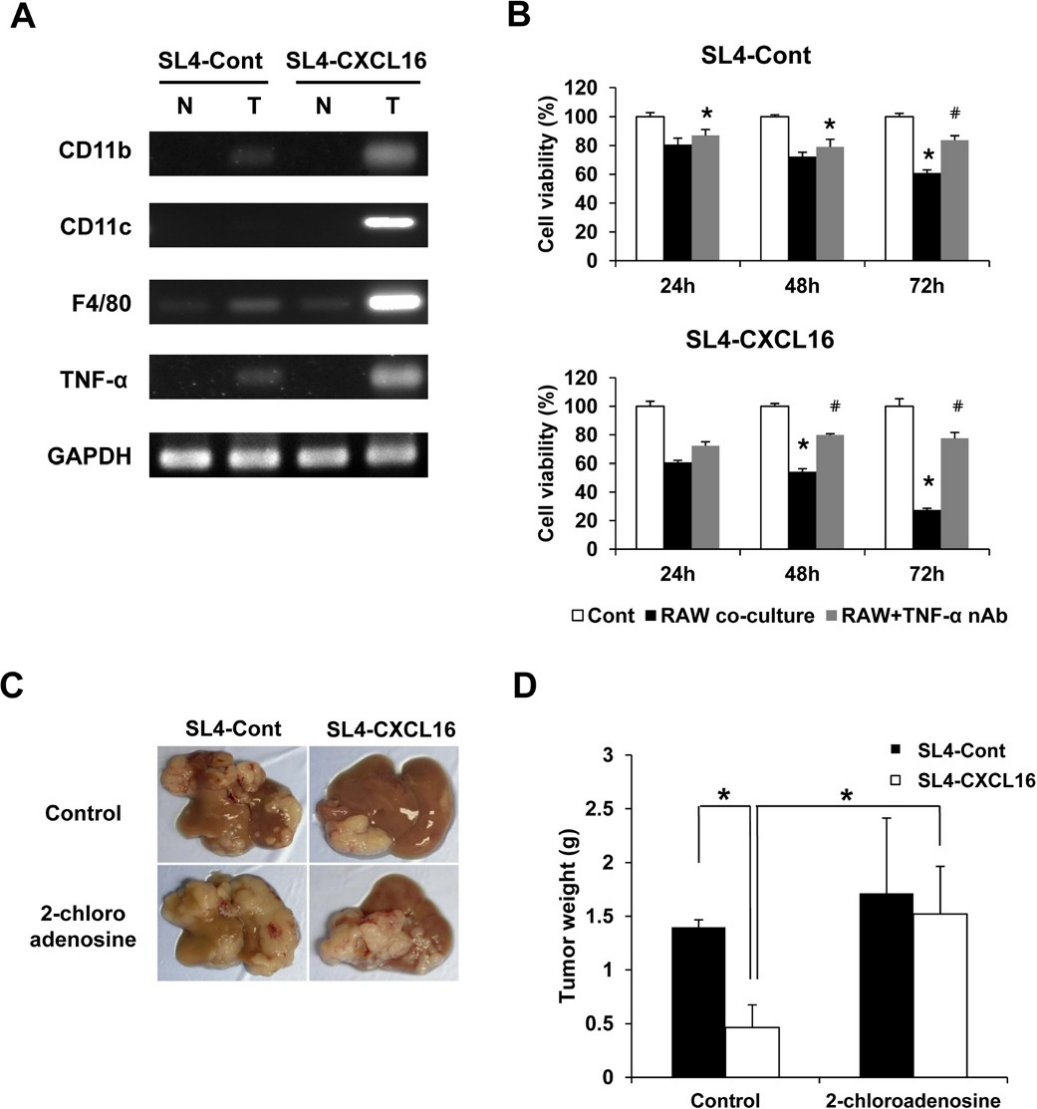
**Effect of 2-chloroadenosine on CXCL16-mediated inhibition of liver metastasis by SL4-CXCL16 cells. (A)** M1 macrophage markers and TNF-α were detected by RT-PCR. GAPDH was used as the normalization control. N, normal; T, tumor. **(B)** Cytotoxicity of macrophage-derived TNF-α and recovery by TNF-α neutralizing antibody in SL4 cells. Cells (5 × 10^4^ cells) were seeded in 24-well plates and a TNF-α neutralizing antibody added (2.5 μg/ml). RAW 264.7 cells (5 × 10^4^ cells) were seeded in migration chambers and co-cultured. Cells were removed from the chambers and their viability was measured by WST-8 assay. *P <0.05, compared with control. ^#^ *P* <0.05, compared with RAW co-culture. **(C** and **D)** Restoration of liver metastasis by CXCL16 expression in a macrophage depletion model. 2-Chloroadenosine was dissolved in saline and injected intraperitoneally (50 μg/100 μl) 24 h before tumor inoculation. *P <0.05. All experiments were repeated at least three times.

CXCL16 has been reported to play an important role in immunosurveillance as a chemoattractant [12, 13]. We determined the mechanism of the inhibition of liver metastasis by CXCL16 by carrying out RT-PCR to identify the types of cells that infiltrated the liver. As shown in Figure 6A, the levels of M1 macrophage markers such as CD11b, CD11c and F4/80 were increased at the tumor sites. We hypothesized that M1 macrophages were recruited and inhibited liver metastasis through the secretion of TNF-α. The M1 phenotype is typically IL-12, TNF-α, IL-6, CXCL9, CXCL10, CXCL11, CXCL16, IL-8^high^, whereas the M2 phenotype is IL-10, CXCR1, CXCR2, CCL17, CCL22, CCL24, CCL16, CCL18, CCL1, CCR2^high^ [26]. Among the M1 and M2 macrophage markers, we selected 10 markers and confirmed in RAW264.7 cells using the RT-PCR method. As a result, we found that phenotype of RAW264.7 cells seem to be M1 type macrophages. Therefore, we conducted a co-culture experiment using RAW264.7 cells as a M1 type macrophage (Figure 6B and Additional files 3 and 4). We confirmed this hypothesis by co-culturing SL4-Cont and SL4-CXCL16 with RAW 264.7 cells and then a TNF-α neutralizing antibody was added to detect differences in cell death. Apoptosis of SL4-CXCL16 was significantly increased by co-culture with RAW 264.7 cells (Figure 6B, middle bar), and neutralization of TNF-α significantly inhibited this response (Figure 6B, right bar). This observation confirmed that CXCL16 expression sensitized the metastatic CRC cell line to apoptosis induced by TNF-α secreted by macrophages. Treatment of mice with 2-chloroadenosine, which is used for macrophage depletion [39], restored liver metastasis (Figure 6C) and also significantly increased tumor weight (Figure 6D). Collectively, these results suggest that CXCL16 expression by SL4 cells induced the accumulation of M1 macrophages, which then induced apoptosis in SL4 cells by secreting TNF-α, thereby leading to inhibition of metastasis.

## Discussion

Chemokines have potential use in cancer gene therapy due to their ability to attract immune cells. However, the metastatic effect of tumor cell-derived CXCL16 on colorectal liver metastasis has not been clarified. The forced expression of CXCL16 in our SL4 subline affected the expression of genes involved in the TNF and apoptosis pathways (Table 1), which provided the first clue to the role of CXCL16 as an intracellular signaling molecule.

IRF8, which was strongly upregulated in SL4-CXCL16 cells (Table 1), has been reported to sensitize Fas-mediated apoptosis in soft tissue sarcoma cells and to modulate the metastatic phenotype of CRC by regulating Fas expression [37, 38]. We confirmed increased IRF8 expression in SL4-CXCL16 cells (Figure 3A) and that knockdown of CXCL16 decreased IRF8 expression in these cells (Figure 3B and C). Conversely, CXCL16 expression was decreased by knockdown of IRF8 in SL4-CXCL16 cells (data not shown), indicating mutual regulation of CXCL16 and IRF8 expression.

Knockdown of IRF8 expression also significantly decreased TNF-α-induced apoptosis in SL4-CXCL16 cells (Figure 4B and C). The Bcl-2 family is regulated by IRF8 in soft tissue sarcoma cells and myeloid cells [40–42], but we found no effect of IRF8 on the Bcl-2 family in SL4 cells (data not shown). Instead, IRF8 appeared to regulate the caspase-3- and PARP-mediated apoptosis pathways following TNF-α treatment (Figure 4). Based on these data, we conclude that CXCL16 upregulates the expression of IRF8, which in turn determines the sensitivity to TNF-α-induced apoptosis. This result provides the first clue in elucidating the role of CXCL16 as an intracellular signaling molecule. Further studies are now needed to determine how the constitutive expression of CXCL16 mediates intracellular signaling.

Soluble CXCL16 is a chemoattractant that induces directed migration of CXCR6-expressing cells [7, 12, 13]. Macrophages do not express CXCR6 and therefore are not attracted by CXCL16. However, CXCL16 positively regulates macrophage accumulation in injured muscle [43], suggesting that an indirect mechanism exists for the attraction of macrophages by CXCL16. Our analysis of the metastatic tumor tissues of SL4-CXCL16 cells indicated the accumulation of NKT cells [44].

Suppression of liver metastasis by CXCL16 expression was partially dampened in NKT cells-depleted mice (data not shown) and infiltration of M1 macrophages was also decreased in these mice (data not shown). NKT cell activation has also been reported following injection of α-galactosylceramide, which induced the infiltration of M1 macrophages and production of TNF-α [45]. Therefore, NKT cells attracted by CXCL16 may have induced M1 macrophage infiltration to the liver and thus indirectly affected liver metastasis through TNF-α production; however, further studies are needed to elucidate the relationship between NKT cells and macrophages.

M1 macrophages produce TNF-α, which has cytotoxic activity against tumor cells [30]. We showed that co-cultured M1 macrophage-like RAW 264.7 cells induced apoptosis in SL4-CXCL16 cells (Figure 6B). Furthermore, liver metastasis by SL4-CXCL16 cells was significantly increased in mice treated with 2-chloroadenosine (Figure 6C and D) to deplete macrophage numbers and depletion of macrophages also reduced TNF-α production (data not shown).

## Conclusions

In conclusion, we have shown that tumor-derived CXCL16 is a key factor in colorectal liver metastasis. Overexpression of CXCL16 sensitizes metastatic CRC cells to TNF-α-induced apoptosis via IRF8. Moreover, CXCL16 also indirectly induces the infiltration of M1 macrophages, which induce tumor cell apoptosis by secreting TNF-α. CXCL16 may be an attractive candidate for gene therapy in colorectal liver metastasis because of its effective dual approach of not only accumulating TAMs, but also increasing cancer cell apoptosis. Although further studies are required to obtain greater insight into the function of this molecule, CXCL16 may have a vital role in the study of cancer immunology and cancer biology.

## Electronic supplementary material

Additional file 1: **Expression of membrane-bound CXCL16 on SL4-CXCL16 cells SL4-CXCL16 cells were incubated with goat anti-mouse CXCL16 mAb (R&D Systems, Minneapolis, MN, USA), or with control goat IgG (R&D Systems).** FITC conjugated rabbit anti-goat IgG (MP Bio Japan, Tokyo, Japan) was used as the second antibody. FITC-labeled cells were then analyzed by flow cytometric analysis using FACSCanto (BD Biosciences, San Diego, CA, USA). FACS profiles by control goat IgG (open area) and anti-mouse CXCL16 mAb (shaded area) are shown. (PDF 90 KB)

Additional file 2: **Expression of CXCR6 on SL4-CXCL16 cells.** SL4-CXCL16 cells were incubated with FITC conjugated rat anti-mouse CXCR6 mAb (R&D Systems, Minneapolis, MN, USA), or with FITC conjugated control rat IgG (R&D Systems). FACS profiles by control rat IgG (black line) and anti-mouse CXCR6 mAb (red line) are shown. (PDF 92 KB)

Additional file 3: **mRNA levels of M1 and M2 macrophage markers in RAW 264.7 cells.** RT-PCR analysis was performed for identification of RAW 264.7 cells phenotype. Briefly, RAW 264.7 cells were seeded in 6-well plates and incubated for 24 h without stimulation. Cells were harvested using scraper and total RNA were extracted. GAPDH was used as the normalization control. The primer sequences are shown in methods and Additional file 4. (PDF 105 KB)

Additional file 4: **Sequences of the RT-PCR primers.** (PDF 70 KB)
