# Supplementary material for: CXCL16 suppresses liver metastasis of colorectal cancer by promoting TNF-α-induced apoptosis by tumor-associated macrophages
Source: BMC Cancer. 2014 Dec 15;14:949. doi: 10.1186/1471-2407-14-949 (PMC4300614; doi:10.1186/1471-2407-14-949)
Supplement: Supplementary file 1 — Additional file 1: Expression of membrane-bound CXCL16 on SL4-CXCL16 cells SL4-CXCL16 cells were incubated with goat anti-mouse CXCL16 mAb (R&D Systems, Minneapolis, MN, USA), or with control goat IgG (R&D Systems). FITC conjugated rabbit anti-goat IgG (MP Bio Japan, Tokyo, Japan) was used as the second antibody. FITC-labeled cells were then analyzed by flow cytometric analysis using FACSCanto (BD Biosciences, San Diego, CA, USA). FACS profiles by control goat IgG (open area) and anti-mouse CXCL16 mAb (shaded area) are shown. (PDF 90 KB) [file 12885_2014_5116_MOESM1_ESM.pdf]

Additional files

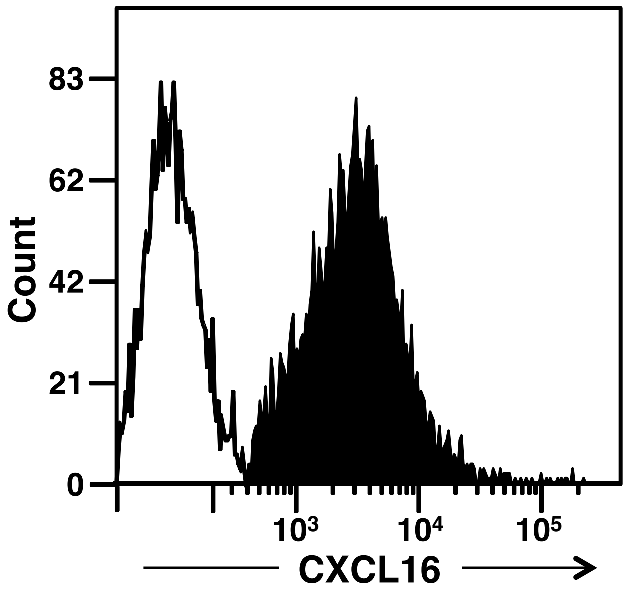

Additional file 1: Expression of membrane-bound CXCL16 on SL4-CXCL16 cells

SL4-CXCL16 cells were incubated with goat anti-mouse CXCL16 mAb (R&D Systems, Minneapolis, MN, USA), or with control goat IgG (R&D Systems). FITC conjugated rabbit anti-goat IgG (MP Bio Japan, Tokyo, Japan) was used as the second antibody. FITC-labeled cells were then analyzed by flow cytometric analysis using FACSCanto (BD Biosciences, San Diego, CA, USA). FACS profiles by control goat IgG (open area) and anti-mouse CXCL16 mAb (shaded area) are shown.
