# Supplementary material for: CXCL16 suppresses liver metastasis of colorectal cancer by promoting TNF-α-induced apoptosis by tumor-associated macrophages
Source: BMC Cancer. 2014 Dec 15;14:949. doi: 10.1186/1471-2407-14-949 (PMC4300614; doi:10.1186/1471-2407-14-949)
Supplement: Supplementary file 2 — Additional file 2: Expression of CXCR6 on SL4-CXCL16 cells. SL4-CXCL16 cells were incubated with FITC conjugated rat anti-mouse CXCR6 mAb (R&D Systems, Minneapolis, MN, USA), or with FITC conjugated control rat IgG (R&D Systems). FACS profiles by control rat IgG (black line) and anti-mouse CXCR6 mAb (red line) are shown. (PDF 92 KB) [file 12885_2014_5116_MOESM2_ESM.pdf]

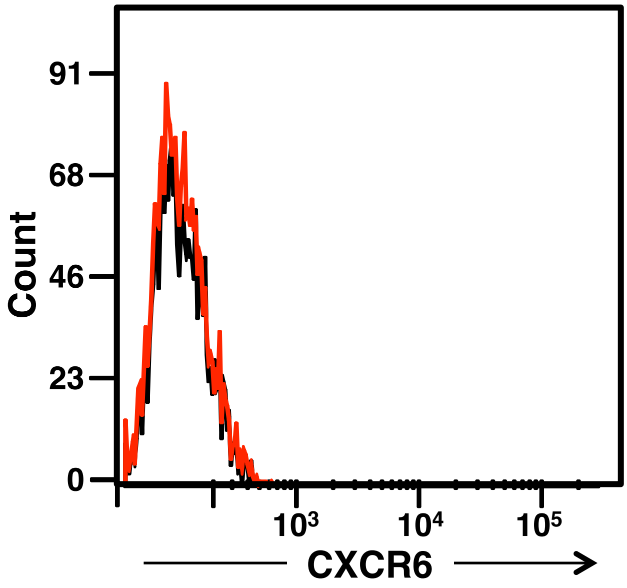

Additional file 2: Expression of CXCR6 on SL4-CXCL16 cells

SL4-CXCL16 cells were incubated with FITC conjugated rat anti-mouse CXCR6 mAb (R&D Systems, Minneapolis, MN, USA), or with FITC conjugated control rat IgG (R&D Systems). FACS profiles by control rat IgG (black line) and anti-mouse CXCR6 mAb (red line) are shown.
