# Supplementary material for: CXCL16 suppresses liver metastasis of colorectal cancer by promoting TNF-α-induced apoptosis by tumor-associated macrophages
Source: BMC Cancer. 2014 Dec 15;14:949. doi: 10.1186/1471-2407-14-949 (PMC4300614; doi:10.1186/1471-2407-14-949)
Supplement: Supplementary file 3 — Additional file 3: mRNA levels of M1 and M2 macrophage markers in RAW 264.7 cells. RT-PCR analysis was performed for identification of RAW 264.7 cells phenotype. Briefly, RAW 264.7 cells were seeded in 6-well plates and incubated for 24 h without stimulation. Cells were harvested using scraper and total RNA were extracted. GAPDH was used as the normalization control. The primer sequences are shown in methods and Additional file 4. (PDF 105 KB) [file 12885_2014_5116_MOESM3_ESM.pdf]

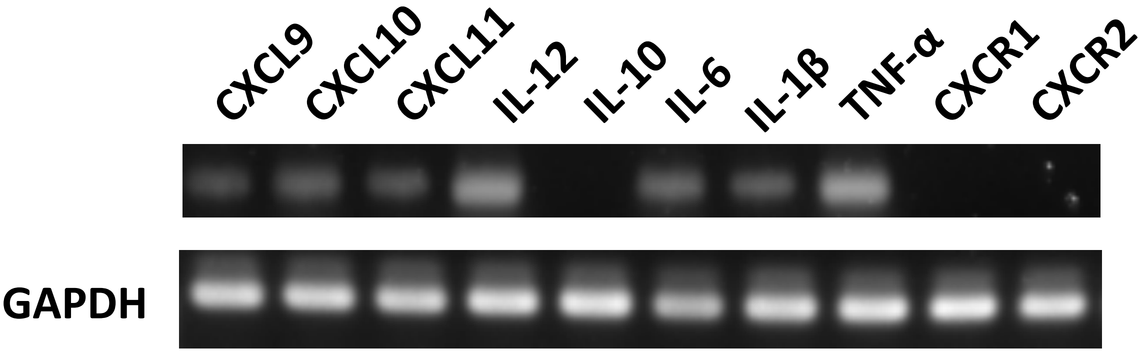

Additional file 3: mRNA level of M1 and M2 macrophage markers in RAW 264.7 cells.

RT-PCR analysis was performed for identification of RAW 264.7 cells phenotype. Briefly, RAW 264.7 cells were seeded in 6-well plate and incubated 24 h without stimulation. Cells were harvested using scraper and total RNA were extracted. GAPDH was used as the normalization control. The primer sequences are shown in methods and additional file 4.
