# Supplementary material for: CXCL16 suppresses liver metastasis of colorectal cancer by promoting TNF-α-induced apoptosis by tumor-associated macrophages
Source: BMC Cancer. 2014 Dec 15;14:949. doi: 10.1186/1471-2407-14-949 (PMC4300614; doi:10.1186/1471-2407-14-949)
Supplement: Supplementary file 4 — Additional file 4: Sequences of the RT-PCR primers. (PDF 70 KB) [file 12885_2014_5116_MOESM4_ESM.pdf]

Additional file 4: Sequences of the RT-PCR primers

Genes	Forward (5’-3’)	Reverse (5’-3’)
CXCL9	CTTTTCCTCTTGGGCATCAT	GCATCGTGCATTCCTTATCA
CXCL10	GCTGCCGTCATTTTCTGC	TCTCACTGGCCCGTCATC
CXCL11	GCTGCTGAGATGAACAGGAA	CCCTGTTTGAACATAAGGAAGC
IL-12	GACTCCAGGGGACAGGCTA	CCAGGAGATGGTTAGCTTCTGA
IL-10	CAGAGCCACATGCTCCTAGA	TGTCCAGCTGGTCCTTTGTT
IL-1β	AGTTGACGGACCCCAAAAG	AGCTGGATGCTCTCATCAGG
CXCR1	TTCTGAGCTTGCTGGGAAAC	GGGTCCTTCGCCTGTATAAGA
CXCR2	CAGGACCAGGAATGGGAGTA	TCCCCTCCAAATATCCCCTA
